# Reliability and Repeatability of ACL Quick Check^®^: A Methodology for on Field Lower Limb Joint Kinematics and Kinetics Assessment in Sport Applications

**DOI:** 10.3390/s22010259

**Published:** 2021-12-30

**Authors:** Annamaria Guiotto, Alfredo Ciniglio, Fabiola Spolaor, Davide Pavan, Federica Cibin, Alex Scaldaferro, Zimi Sawacha

**Affiliations:** Department of Information Engineering (DEI), University of Padova, 35131 Padova, Italy; guiotto@dei.unipd.it (A.G.); ciniglioal@dei.unipd.it (A.C.); fabiola.spolaor@unipd.it (F.S.); davide.pavo.pavan@gmail.com (D.P.); fede.cibin90@gmail.com (F.C.); alexscaldaferro@gmail.com (A.S.)

**Keywords:** joint moments, ACL injury, video analysis, plantar pressure, sagittal plane kinematics of squat

## Abstract

Anterior cruciate ligament (ACL) lesion represents one of the most dramatic sport injuries. Even though clinical screenings aiming at identifying subjects at risk of injuries are gaining popularity, the use of sophisticated equipment still represents a barrier towards their widespread use. This study aimed to test both reliability and repeatability of a new methodology to assess lower limb joints kinematics and kinetics directly on field with the aid of video cameras and plantar pressure insoles. Ten athletes and one case study (post ACL surgery) were assessed in a gait laboratory, while performing double leg squats, through the simultaneous acquisition of stereophotogrammetry, force plates, commercial video cameras and plantar pressure insoles. Different sources of errors were investigated and both reliability and repeatability analysis performed. Minimum and maximum RMSE values of 0.74% (right knee joint center trajectory) and 64.51%, respectively (ankle dorsi-plantarflexion moment), were detected. Excellent to good correlation was found for the majority of the measures, even though very poor and inverse between-trials correlation was found on a restricted number of trials especially for the ankle dorsi-plantarflexion moment. These findings could be used in combination with already available screening tools in order to provide more repeatable results.

## 1. Introduction

Anterior cruciate ligament (ACL) ruptures represent one of the healthcare emergencies among sport injuries and have received much attention in recent years [1,2,3]. ACL injury can be the cause of long-term disability and is one of the most frequent causes of loss of a sport season [4,5]. It is estimated that the incidence of ACL ruptures range from 30 to 78 per 100,000 person-years, and 61–83% of athletes successfully return to sports [2,6] typically 8–18 months after reconstruction and most of them perform similarly to uninjured athletes when compared at competing level [2]. ACL injuries can occur from both non-contact and contact sports: among the noncontact sports, the most commonly cited are soccer, basketball, alpine skiing and gymnastics, while for the contact sports the highest injury rates are reported for football, rugby, wrestling and ice hockey [4]. From a biomechanical point of view, in the case of ligament injuries, the purpose of preventive strategies is aimed at reducing the stress placed through the ligament; moreover, in the case of reinjury, when the ligament, not necessarily, can withstand the same stress before failure [2].

In order to understand the etiology of ACL rupture, we should consider that ACL consists of two major bundles, the posterolateral and the anteromedial bundles, that originate on the posteromedial side of the lateral femoral condyle and insert on a region just anterior to the intercondylar tibial eminence [7,8]. The main role of the ACL is to control anterior movement of the tibia and to inhibit extreme ranges of tibial rotation, hence knee motion beyond the normal physiological ranges in any plane may result in ligamentous injury [9,10].

Indeed, the majority of ACL noncontact injuries occur during landing or during cutting maneuvers, when either keeping the knee in the valgus position deep flexion or both [11,12], and there is evidence that the frequent adoption of deep squat movements may represent a risk factor for collateral and ACL injuries [12].

Based on these considerations, evaluating lower extremity kinematics during deep squatting becomes relevant when addressing the topic of knee injuries, their prevention and their rehabilitation [4,13,14,15]. In fact, differences in lower extremity kinematics has been detected when comparing ACL-repaired athletes with non-injured controls [12,16]. As a consequence, it is now common for sport clinicians to screen individuals for dynamic lower extremity postures during functional movements, such as squats.

In this context, it is worth noticing that a large variety of functional screening tools are available in the literature and among them the most commonly used are the following: Functional Movement Screen, Star Excursion Balance Test, Y Balance Test, Drop Jump Screening Test, Landing Error Scoring System and the Tuck Jump Analysis [17,18,19,20,21,22].

Generally, they imply visual scoring of different movements as well as pain, and the best score is associated with the ability to correctly complete the movement without compensation. In some cases, measuring of reaching distances marked on the floor through strips of tape can be involved (Star Excursion Balance Test). Among them the LESS appeared to be the most reliable tool validated against three-dimensional motion analysis even though some items/errors have not been completely validated, and sex, fatigue and previous ACL rupture have demonstrated to affect LESS performance [23]. In this respect, validation of the optimal cut-point to predict injury risk for all these tests is still lacking [24].

Besides these functional tests, athletes are usually assessed with respect to their performance during some fundamental movements including running, throwing, jumping and squatting [25]. Among these, the squat movement is required in most sport activities and can also be linked to other activities of daily living, such as sitting and lifting [26]. It is also a fundamental exercise used to train athletic performance across different sports thanks to its ability to promote coordination of numerous muscle groups while strengthening the ones necessary to build quick, explosive power and decrease the risk of training induced injuries [27]. For these reasons, it has been proposed for use as a screening tool; on one side to assess the neuromuscular control, strength and stability, on the other side to identify the biomechanical deficits and their neuromuscular implications that may compromise performance and injury resilience [26].

Regardless of the numerous tests and research works that evaluated physical performance tests at the knee, evidence for their measurement quality is still limited and results are contradictory [24,28,29]. Given the difficulties in reporting reliable and repeatable visual scoring, there is now a tendency to incorporate 2-D motion analysis software applications that are increasingly available [11]. However when sophisticated and research equipment are needed, in the majority of cases, this represents a barrier to using these screening methods in clinical practice during preseason physical examination [4,20,21]. To the best of the authors’ knowledge [4], with the adoption of motion analysis equipment, no studies were conducted with the purpose of assessing athletes for deciding upon their return to the field. This is mainly associated with the complexity and the costly nature of this equipment. Furthermore, even though inertial measurement systems have gained popularity in the assessment of athletes’ performances [24], there is a paucity of works reporting on their use in prevention screenings [4].

In the context of motion analysis application to ACL injury risk prevention, a methodology based on plantar pressure insoles and video cameras was recently developed and patented by the authors at the University of Padova, under the name of “ACL Quick Check^®^” (No. 302017000089339, August 2017), with the aim of providing a quantitative screening for ACL injury risk applicable on the field (patent No. 102017000046512). Given that the majority of ACL noncontact injuries occur when either keeping the knee in the valgus position, deep flexion or both [12], the present methodology allows assessing lower limb (hip, knee, ankle) sagittal plane kinematics, flexion-extension and abduction-adduction moments. This is in agreement with the majority of the literature adopting motion analysis for ACL injury screening [30,31]. In order to reduce both costs and complexity of use associated with the adoption of motion analysis techniques, for the assessment of kinematics and kinetics, respectively, stereophotogrammetry was substituted with commercially available video cameras, and force plates were substituted with plantar pressure insoles.

The aim of this paper was to test both reliability and repeatability of this methodology with respect to similar measures provided by stereophotogrammetry and force plates as a gold standard. To this end, a group of athletes was assessed in a gait laboratory, while performing a series of double leg squats, through the simultaneous acquisition of a stereophotogrammetric system, two force plates, commercial video cameras and plantar pressure insoles. The major sources of errors were investigated through the comparison between kinematics and kinetics variables estimated by means of ACL Quick Check^®^ and standard motion analysis techniques (i.e., stereophotogrammetry and force plates). Nevertheless, errors in estimating marker trajectories on one side through video-based analysis, and on the other one ground reaction forces through plantar pressure insoles were assessed. Root mean square error (RMSE), coefficient of multiple correlation (CMC) and Pearson correlation coefficient were calculated [23,25]. As ACL Quick Check^®^ implies tracking of video sequences, inter-rater reliability was also assessed [32] to verify the ability of the system to accurately track markers.

## 2. Materials and Methods

### 2.1. Subjects

The dataset reported in this study is a retrospective analysis of data available within the database of BiomovLab (Department of Information Engineering of the University of Padova), related to squat tasks collected from two cohorts of athletes who participated in studies exploring the biomechanics of multiple tasks (i.e., gait, jumping, drop landing, squat). The study, from which data were selected, was conducted according to the guidelines of the Declaration of Helsinki, and approved by the Ethics Committee of Azienda Ospedaliera Padova (Protocol No. 3493/AO/15). A priori sample size calculations, based on previous publications [33,34], were conducted; assuming a significance level (α) = 0.05, probability of type II error (β) = 0.2, acceptable reliability (ρ0) = 0.5 and expected reliability (ρ1) = 0.8, a sample size of 8.2 subjects was necessary. Hence, 10 subjects was deemed sufficient for the present study. Inclusion criteria for the healthy subjects’ data were: available signed informed consent, athletes competing at professional or amateur level, no previous lower limb injuries, absence of neurological pathologies associated with any musculoskeletal disease, availability of double leg squat tasks simultaneously acquired through stereophotogrammetry, force plates, plantar pressure sensors and video cameras. 

Based on these criteria the data of ten healthy subjects (HS), five males and five females, were considered for the present study and their demographic and anthropometric characteristics reported in Table 1. Furthermore the data of one subject who suffered an ACL rupture acquired 4 months after the ACL surgery reconstruction (i.e., semitendinosus and gracile) [35,36] were included as a case study in Appendix A. Anonymized data were available and each subject was associated with a numerical code. Each subject performed 3 double leg squats, thus the database included the data of 30 trials.

### 2.2. Instrumental Protocol

**Figure 1:**
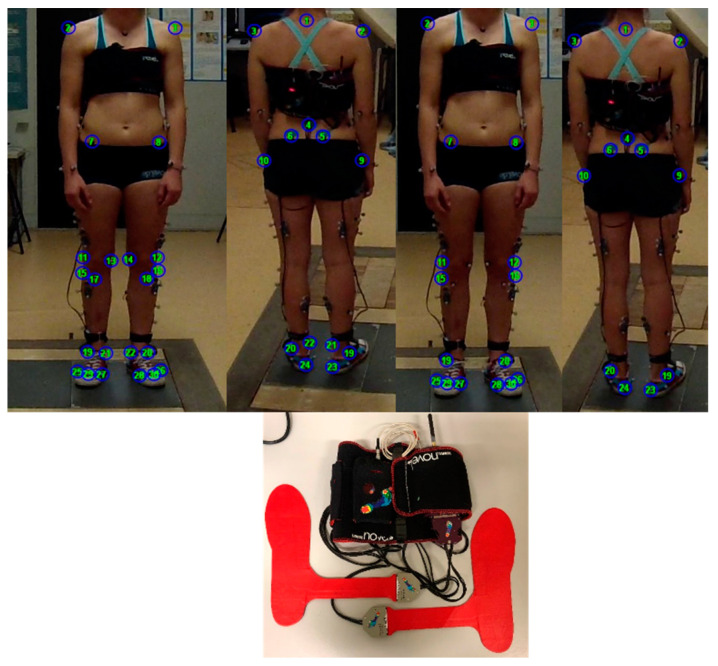
Top: IORgait marker set as visible through Track On Field software; bottom: the plantar pressure insole system.

All the data were simultaneously acquired at the BiomovLab (Department of Information Engineering of the University of Padova), through a stereophotogrammetric system (BTS, 6 cameras, 60 Hz) [37,38], synchronized with two force platforms (Bertec FP4060, 960 Hz) [39], a plantar pressure insoles system (Pedar-X, Novel, 100 Hz) [40,41] and eight commercial cameras (four GoPro Hero3 and four GoPro Hero7, 1080 × 1920 pixel resolution, 30 fps). Appendix B was added to report the technical datasheet of the instruments. The data of the plantar pressure system and the force plates were synchronized in post processing by considering the first instant of the center of pressure (COP) measure, while the data of the commercially available system and the motion capture system were synchronized by considering the 5th lumbar vertical marker trajectory. Reflective markers were applied on each subject according to the IORgait marker set [42] (Figure 1, Appendix C). Previous research has demonstrated the reliability of this model in an adult population [43,44,45]. 

**Figure 2:**
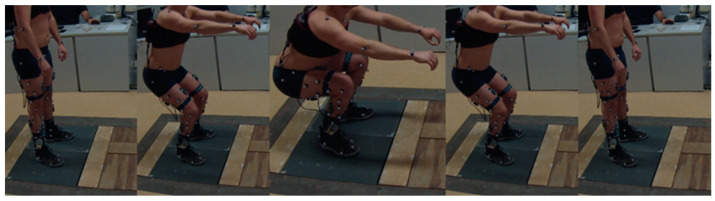
Stages of the bipodalic squat (left to right): (1) starting point, (2) descending phases, (3) peak flexion, (4), ascending phases, (5) return to starting point.

All the subjects were instructed to perform the squat task with feet placed shoulder width apart, arms stretched out in front of the body and parallel to the floor and according to the following instruction: “descend to a level to where your posterior thighs are parallel to the floor and then ascend” [46] (Figure 2). Every subject performed 3 double leg squats.

### 2.3. Data Analysis

Once acquired, the video sequences were processed in order to extract the 3 dimensional (3D) anatomical landmark coordinates through “Track On Field” software ((TOF) BBSoF S.r.l.) (Figure 1), validated in [47]. From both the force plates and the plantar pressure sensors the COP was extracted together with the 3D or 1D ground reaction forces (GRFs), respectively. The data were filtered by adopting similar filters for signals related to kinematic and kinetic parameters when involved in the calculation of joint moments, according to [48]. Both the motion capture and TOF data were filtered with a th-low pass Butterworth filter (6 Hz cut off frequency) [49] and similarly the force plate and plantar pressure data were filtered with a 4th order low pass Butterworth filter (6 Hz cut of frequency) which concerns the GRF and with a 5th order low pass Butterworth filter (7 Hz cut of frequency) which concerns the COP [50]. Hence the following variables were determined:

- hip, knee and ankle joints’ sagittal plane kinematics determined according to [42,51,52,53]
- hip, knee, ankle abduction-adduction and flexion extension moments considering two different marker sets, the ACL Quick Check^®^ with the complete IORgait marker set [42] (referred to as ACL Quick Check^®^ integrated in the following paragraphs) and the ACL Quick Check^®^ with a simplified marker set as in [47]. Differently from the original publication [42], in both versions the hip joint center was determined according to the regression equations proposed by Harrington et al. (2007) [54]
- hip, knee, ankle abduction-adduction and flexion extension moments considering the ground reaction vector technique [55,56,57,58]
- hip, knee, ankle abduction-adduction and flexion-extension moments considering the inverse dynamic approach which follows the Newton–Euler method [49,56,59].

Details on joint moment calculations are reported in Appendix C.

In order to reduce the effect of a temporal shift due to a contraction or dilation of the squat duration, a time normalization was performed on each variable with respect to the duration of the squat cycle identified by considering the 5th lumbar vertebra trajectory, from the first instant of descending to the instant in which the starting position was reached again. Furthermore, GRFs were normalized according to body weight and joint moments, defined as external moments, were normalized to body weight times height. All the analyses were done in (Matlab R2019b, MathWorks).

For reporting the data on the figures, the average and standard deviation among right and left limb data was calculated, the average was reported as a solid line and the band created with the average plus and minus one standard deviation was plotted as colored transparent filled band.

#### 2.3.1. Reliability Analysis

To assess reliability of the proposed methodology, the different sources of errors were considered according to [56], specifically: kinematic measures and processing, measure of the GRF and processing, determination of joint model parameters. These were classified as extrinsic (i.e., measurement errors) and intrinsic (i.e., the choice of the coordinate system to report intersegmental moments, the choice of the dynamics equations) sources of variability. 

In order to assess the impact of the extrinsic sources, the following comparisons were made:

- 3D joint centers trajectories computed by means of stereophotogrammetry vs. video based (TOF). This comparison aims to identify the influence of differences in the estimation of the joint centers’ trajectories on joint angles and intersegmental moments, given that joint center positions are used either to define the moment arm of the forces acting on the segment or segment length that are involved in the assessment of body segment inertial parameters [49].
- sagittal plane kinematics based on the marker trajectories reconstructed through stereophotogrammetry vs. the ones reconstructed through TOF.
- vertical component of GRF recorded through the force plates vs. the one retrieved through the plantar pressure system.
- COP displacement recorded through the force plates vs. the one retrieved through the plantar pressure system. In order to compare the two COP paths a rigid transformation was applied to define the COP coordinates of the plantar insoles with respect to the force plates reference system [60].

In order to assess the impact of the intrinsic sources, the following comparisons were made:

e. flexion-extension and abduction-adduction joint moments retrieved according to inverse dynamics procedures (Newton–Euler) [60] by considering the markers’ trajectories reconstructed through stereophotogrammetry combined with the GRF from the force plate, ACL Quick Check^®^ integrated
f. flexion-extension and abduction-adduction joint moments retrieved according to the Ground Reaction Vector approach, which consists of multiplying the ground reaction force vector by its moment arm at each joint (Ground Reaction Vector 3D) [48] ACL Quick Check^®^ integrated. In this case Ground Reaction Vector 3D was applied by considering the markers’ trajectories reconstructed through stereophotogrammetry.
g. flexion-extension and ab- and adduction joint moments retrieved according to the Ground Reaction Vector approach but considering only the vertical component of the GRF (Ground Reaction Vector 1D) and the markers trajectories reconstructed through stereophotogrammetry vs. ACL Quick Check^®^ integrated
h. ACL Quick Check^®^ integrated applied by considering the data acquired with the stereophotogrammetric system and the plantar pressure system (ACL Quick Check^®^ integrated STF) vs. ACL Quick Check^®^ integrated applied by considering the data acquired with commercial video cameras and the plantar pressure system.
i. ACL Quick Check^®^ integrated vs. ACL Quick Check^®^

All the analyses were done in (Matlab R2019b, MathWorks). The following indexes were computed in order to assess the reliability of the measures with respect to the gold standard (i.e., either stereophotogrammetry, force plates or both): RMSE% of the gold standard (i.e., stereophotogrammetry or force plate) in all cases apart from the last comparison (i.) where RMSE was expressed as a percentage of ACL Quick Check^®^ integrated; Pearson’s correlation coefficient (r), after evidence of normality (Lilliefors test) or Spearman’s correlation coefficient. In particular, the correlation coefficient was calculated either between pairs of single values (i.e., COP range and total length in medial-lateral and anterior-posterior directions), or between pairs of different time series (i.e., when the correlation between measures obtained by different instruments or approaches was addressed) [31,47]. A significant correlation was considered the one with the *p*-value less than 0.05.

Based on previous publications [31,47], the values of r were interpreted as follows:

0.65–0.75: moderate
0.75–0.85: good
0.85–0.95: very good
0.95–1: excellent

#### 2.3.2. Repeatability Analysis

By considering errors in marker tracking will directly impact on the estimation of the 3D markers’ trajectories and consequently on the joint centers; the inter-rater repeatability was assessed on 3D hip, knee and ankle joints’ center coordinates and on flexion-extension angles. Three raters (with at least 1 year of experience) performed the automatic tracking of features of 3 double leg squat tasks from the same subject, and CMC [32] was computed across the different measures. The values of CMC were interpreted as described in the preceding paragraph with respect to Pearson’s correlation. All the analyses were done in (Matlab R2019b, MathWorks).

## 3. Results

**Figure 3:**
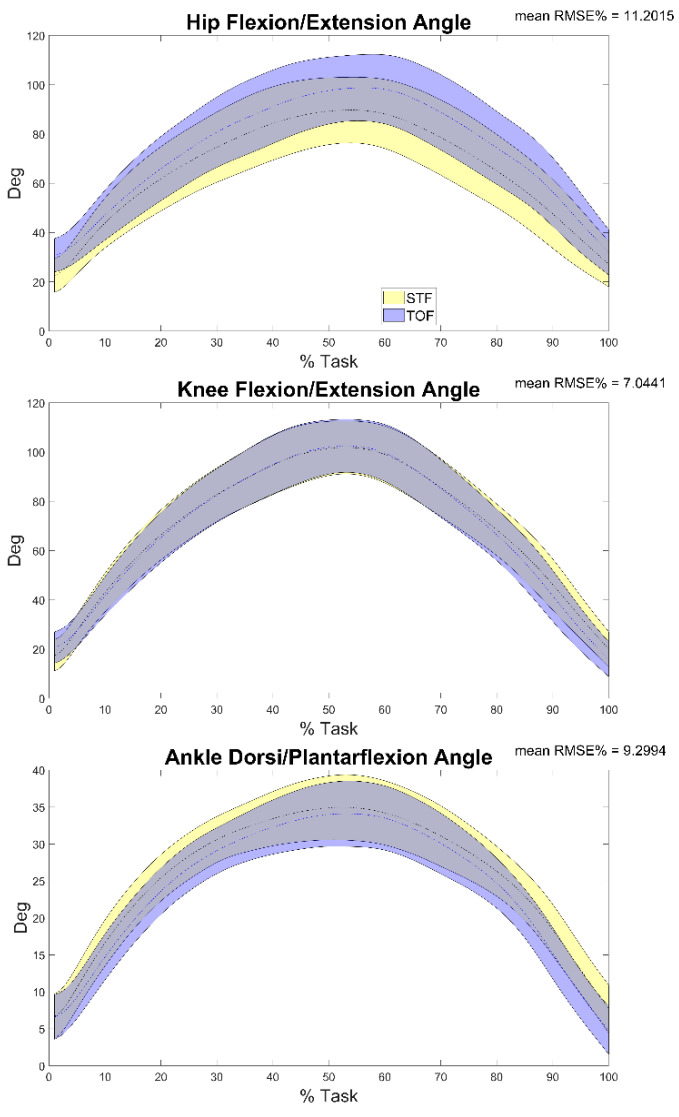
Hip, knee and ankle flexion/extension angles during the task calculated through stereophotogrammetry (STF—blue) and video-based tracker (TOF—yellow) and the mean RMSE% of the comparison between the two. Averages plus and minus one standard deviation are reported as colored bands.

**Figure 4:**
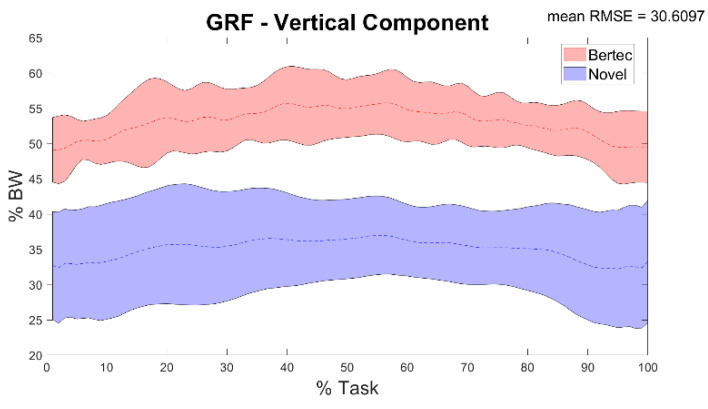
Vertical component of the GRF during the task recorded through the force plates (Bertec—red), and through the plantar pressure system (Novel—blue) and mean of RMSE% of the comparison between the two. Averages plus and minus one standard deviation are reported as colored bands.

**Figure 5:**
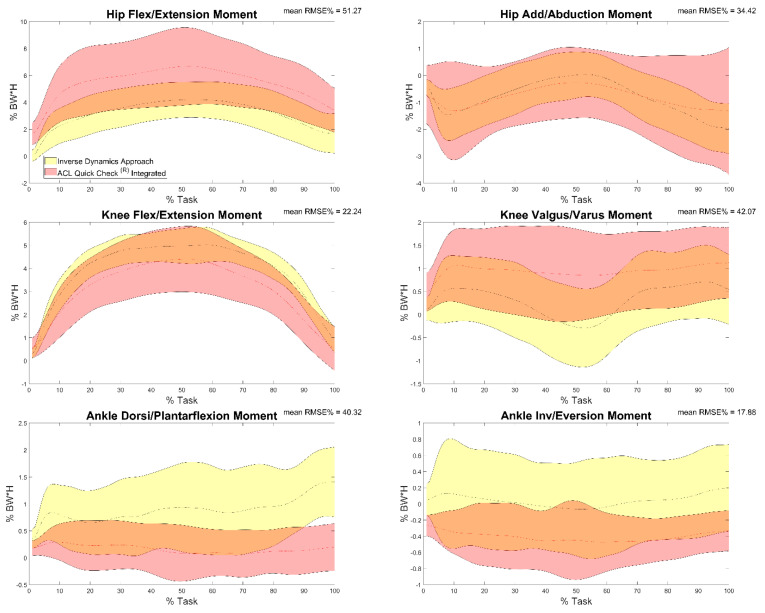
Hip, knee and ankle adduction/abduction and flexion/extension moments during the task calculated through inverse dynamics (yellow) and through ACL Quick Check^®^ integrated (red) and mean of RMSE% of the comparison between the two. Averages plus and minus one standard deviation are reported as colored bands.

**Figure 6:**
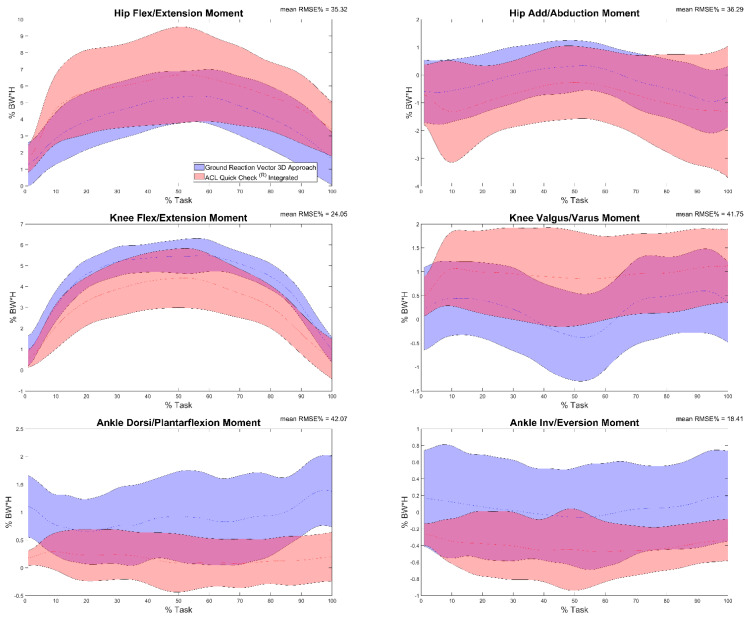
Hip, knee and ankle adduction/abduction and flexion/extension moments during the task calculated through the Ground Reaction Vector 3D approach (blue) and through ACL Quick Check^®^ integrated (red) and mean of RMSE% of the comparison between the two. Averages plus and minus one standard deviation are reported as colored bands.

**Figure 7:**
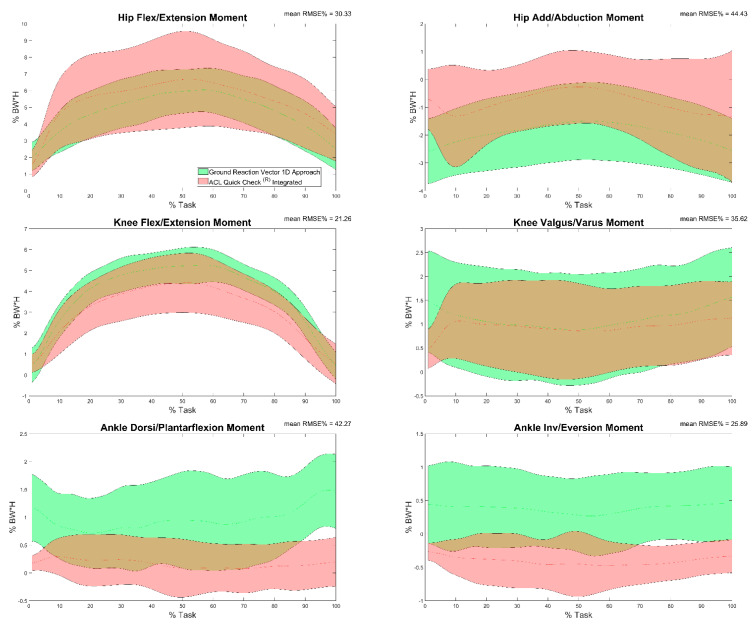
Hip, knee and ankle adduction/abduction and flexion/extension moments during the task calculated through moment arm 1D approach (green) and through ACL Quick Check^®^ integrated (red) and mean of RMSE% of the comparison between the two. Averages plus and minus one standard deviation are reported as colored bands.

**Figure 8:**
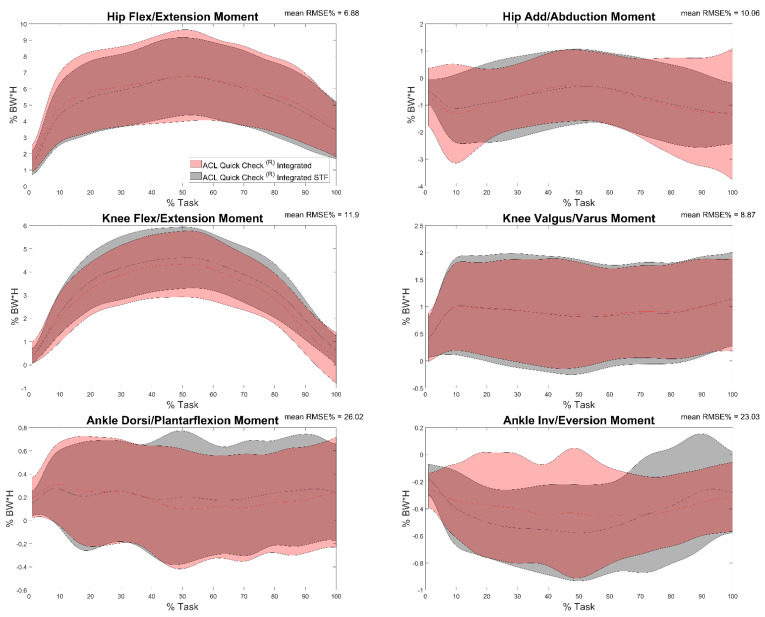
Hip, knee and ankle adduction/abduction and flexion/extension moments during the task calculated through ACL Quick Check^®^ integrated by considering the data acquired with the stereophotogrammetric system (gray) and through ACL Quick Check^®^ integrated by considering the data acquired with commercial video cameras (red) and mean of RMSE% of the comparison between the two. Averages plus and minus one standard deviation are reported as colored bands.

**Figure 9:**
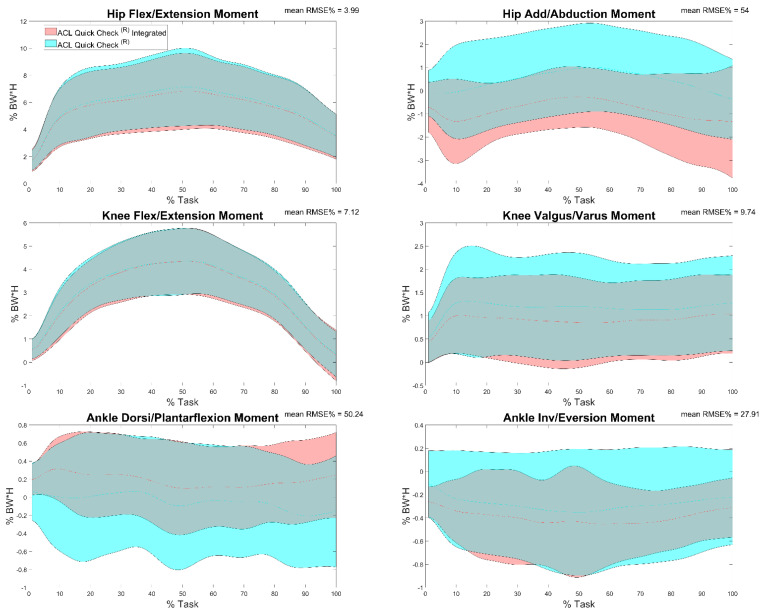
Hip, knee and ankle adduction/abduction and flexion/extension moments during the task calculated through ACL Quick Check^®^ (cyan) and through ACL Quick Check^®^ integrated (red) and mean of RMSE% of the comparison between the two. Averages plus and minus one standard deviation are reported as colored bands.

A total of 30 double leg squat trials were processed for the HS group. Descriptive statistics were reported including mean and standard deviation over a squat cycle (Figure 3, Figure 4, Figure 5, Figure 6, Figure 7, Figure 8 and Figure 9).

### 3.1. Reliability of the Proposed Approach

In the current section results on the reliability of the proposed approach are reported by considering the extrinsic and intrinsic different sources of error.

In Table 2 results on the comparison between joint center trajectories computed based on the markers reconstructed with stereophotogrammetry and those reconstructed through TOF are reported (comparison a). The minimum RMSE value of 0.74% was registered on the Y component of the right knee joint center and the maximum RMSE value of 61.84% was registered on the Y component of the left ankle joint center. In terms of between-trials correlation, excellent to good correlation was shown for the majority of the joint centers with r values ranging from 0.96 to 1. In some cases, a very poor correlation or an inverse correlation was found between a restricted number of trials for the following joint centers: right and left hip joint centers (medial lateral coordinate: −0.46 < r < 1 negative for the 20% of trials, anterior posterior coordinate: 0.27 < r < 1), right and left ankle joint centers (medial lateral 0.26 < r < 0.99 and vertical trajectories −0.1 < r < 0.96—negative for only one trial).

Results of the comparison between the sagittal plane kinematics based on the marker trajectories reconstructed through stereophotogrammetry vs. the ones reconstructed through TOF (comparison b) are reported in Figure 3 and Table 2. Results showed that the minimum RMSE value of 4.13% was registered on the knee flexion/extension angle and the maximum one (15.59%) on the ankle flexion/extension. In terms of r, moderate to excellent correlation was found between trials, with a minimum value of 0.77 detected in correspondence of the hip flexion/extension angle and a maximum value of 1 detected in correspondence of all the joint angles.

In Table 3 and Figure 4 results of the comparison between the vertical component of GRF recorded through the force plates and the plantar pressure system (comparison c) were reported. Minimum and maximum RMSE values were respectively 27.71% and 32.6%, while between-trials correlation varies from very good to very poor with maximum and minimum r values respectively of 0.86 and 0.11.

Results of the comparison between the COP displacement recorded through the force plates and the plantar pressure system (comparison d), were reported in Table 4 and showed the minimum RMSE value on the anterior/posterior COP range (24.6%) and the maximum RMSE value on the medial/lateral COP length (35.84%). Between-trials correlation analysis revealed excellent to poor correlation with maximum and minimum r values registered respectively on the medial/lateral COP standard deviation (0.96) and on the anterior/posterior COP length (0.3).

Results of the comparison between joint moments calculated by means of inverse dynamics procedures and ACL Quick Check^®^ integrated (comparison e) are reported in Table 5 and in Figure 5. Results showed respectively minimum and maximum RMSE values in correspondence with the knee flexion/extension moment (8.25%) and the hip abduction/adduction moment (59.45%). In terms of between-trials correlation analysis, excellent (r = 0.99, maximum value for the knee flexion-extension moment) to poor correlation (r = 0.49, minimum value for the hip flexion-extension moment) was found, with some comparison revealing an inverse correlation (57% of trials for the ankle dorsi-plantarflexion moment, 22% of trials for both knee varus-valgus and ankle inversion-eversion moments).

Results of the comparison between joint moments calculated by means of Ground Reaction Vector 3D and ACL Quick Check^®^ integrated (comparison f) are reported in Table 5 and in Figure 6. Results showed minimum and maximum RMSE values on the knee flexion/extension moment (13.21%) and on the ankle dorsi/plantarflexion moment (64.51%), respectively. Excellent (r = 0.99, maximum value for the knee flexion-extension moment) to very poor correlation (r = 0.31, minimum value for the hip flexion-extension moment) was found between trials, with some comparisons revealing an inverse correlation (57% of trials for the ankle dorsi-plantarflexion moment, 30% of trials for the knee varus-valgus moment and 24% of trials for both ankle inversion-eversion and hip ab- and adduction moments).

Results of the comparison between joint moments calculated by means of Ground Reaction Vector 1D (considering only the vertical component of the GRF from the force plate) and ACL Quick Check^®^ integrated (comparison g) are reported in Table 5 and in Figure 7. Minimum and maximum RMSE values were registered on the knee flexion/extension moment (13.25%) and on the ankle dorsi/plantarflexion moment. Excellent (r = 0.99, maximum value for the knee flexion-extension moment) to very poor correlation (r = 0.46, minimum value for the hip flexion-extension moment) was found between trials, and in some cases an inverse correlation was also detected (60% of trials for the ankle dorsi-plantarflexion moment, 28% of trials for the knee varus-valgus moment, 35% of trials for the ankle inversion-eversion moment and 22% of trials for the hip ab- and adduction moment).

Results of the comparison between joint moments calculated by means of ACL Quick Check^®^ integrated and ACL Quick Check^®^ integrated STF (comparison h) are reported in Table 5 and in Figure 8. Minimum and maximum RMSE values were detected in correspondence of the hip flexion/extension moment (4.73%) and the ankle flexion/extension moment (36.17%), respectively. Excellent (r = 0.9, maximum value for the knee flexion-extension moment) to very poor correlation (r = 0.35, minimum value for the hip flexion-extension moment) was found between trials, and in some cases an inverse correlation was also detected (27% of trials for the ankle dorsi-plantarflexion moment, 22% for the hip add-abduction moment, 16% of trials for the ankle inversion-eversion moment and only one trial for the knee varus-valgus moment).

Results of the comparison between joint moments calculated by means of ACL Quick Check^®^ integrated and ACL Quick Check^®^ (comparison i) are reported in Table 5 and in Figure 9. Minimum and maximum RMSE values were registered on the hip flexion/extension moment (1.54%) and on the ankle flexion/extension moment (62.67%). Excellent (r = 1, maximum value for all the joint moments with the exception of the hip ab- and adduction and the knee varus-valgus moments that displayed a maximum r of 0.94 and 0.99, respectively) correlation was found between the majority of the trials, even though in some cases an inverse correlation was detected (37% of trials for the ankle dorsi-plantarflexion moment, 24% for the hip ab- and adduction moment, 19% of trials for the ankle inversion-eversion moment and only one trial for the knee varus-valgus moment).

### 3.2. Inter-Rater Repeatability

In Table 6 results of the inter-rater repeatability analysis for the joint centers trajectories computed with TOF are reported. Excellent repeatability was found with lowest and highest CMC values respectively of 0.9683 (right hip joint center coordinates on the medial-lateral axis) and 0.9709 (left knee joint center coordinates on the vertical axis). RMSE values (expressed as percentage of the trajectories reconstructed by the most expert rater) ranged between 0.36% (knee joint center coordinates on the medial-lateral axis) and 12.4% (hip joint center coordinates on the anterior-posterior axis).

In Table 6 results of the inter-rater repeatability analysis for the sagittal plane kinematics based on markers trajectories reconstructed through TOF were reported. Excellent repeatability was found for all measures with respectively lowest and highest CMC values of 0.962 (right hip flexion-extension angle) and 0.9834 (right knee flexion-extension angle). RMSE values (expressed as percentage of the rotations reconstructed by the most expert rater) ranged between 1.36% (left knee flexion-extension angle) and 26.74% (left ankle flexion-extension angle).

## 4. Discussion

The effect of all potential mechanisms involved in contact and non-contact lower limb injuries is still poorly understood [23], and for this reason the literature proposed several clinical movement screening tests to analyze these mechanisms [17,18,19,20,21,22]. By considering the mostly unpredictable nature of contact injuries, due to the external mechanism involved, most preseason movement screening tests aim to predict noncontact injury risk and guide injury prevention programs [23]. The costly nature, the complexity of use and the requirement of a controlled environment have prevented the widespread use of these technologies for clinicians’ physical examinations [23]. As a result, clinician-friendly screening tests were developed as an alternative. However, results of their reliability and repeatability are still contradictory [29] and none of the variables within the various screening tests, to the best of the authors’ knowledge, has shown to be able to distinguish athletes who sustained a future noncontact ACL injury from the athletes who remained uninjured [29]. The methodology proposed herein aims at providing a repeatable and reliable quantitative assessment of the biomechanical factors associated with lower limb injury risk, while overcoming the difficulties of employing standard motion analysis techniques within the preseason clinical physical examination screenings. To assess the reliability of the proposed methodology, the different sources of errors were explored starting from the so called “extrinsic sources of errors” associated either with tracking of markers by means of video technologies, or with measures of both the vertical component of the GRF and the COP by means of plantar pressure insoles. With respect to the first one, results of this analysis revealed excellent repeatability in agreement with [47], and very low RMSE% values when compared with stereophotogrammetry. Good results were found in terms of RMSE values, when considering the comparison between plantar pressure insoles and force plates in terms of GRF and COP measures. Correlation analysis results revealed excellent (COP SD medial-lateral direction) to very poor (COP length in anterior-posterior direction) correlation for the COP and very good to very poor correlation for the GRF. When observing these results, it should be considered that sensor insoles measure pressure and the “normal” force, which is generally compared with the vertical ground reaction force from the force plate [61,62]. Even though there are several studies reporting the repeatability and validity of sensor insoles [63,64,65], they only analyzed walking or running tasks. When assessing motion involving shear forces at a larger extent than walking, this may determine an underestimate of the vertical GRF [66]. In the present contribution, the Pedar mobile system (Novel GmbH, Munich, Germany) was used, which is considered a valuable gold standard among plantar pressure insoles systems [63,67,68], however, this is the first time that the normal force derived from this system was compared with the vertical component of the GRF during a squat task.

In analyzing the so-called “intrinsic sources of errors” associated with the procedures adopted for the joint moment calculation, results indicate that the largest errors occurred in association with the exclusion of the anterior-posterior and medial-lateral components of the GRFs from the dynamic equation. Indeed, the comparison between both the inverse dynamics approach or Ground Reaction Vector 3D with ACL Quick Check^®^ (comparisons e and f) revealed the larger number of trials reporting an inverse correlation (a maximum number of 57% of trials was identified for the ankle dorsi-plantarflexion moment). It is worth mentioning that even for these comparisons, every joint moment displayed excellent correlation results for a certain number of trials (maximum r values for each joint were higher than 0.88).

Nevertheless, when considering the sole impact of the plantar insoles in determining the vertical component of the GRF, results indicated a large number of trials displaying an inverse correlation (a maximum number of 60% trials was identified for the ankle dorsi-plantarflexion moment). Furthermore, in this case each joint moment revealed a certain number of trials which displayed an excellent correlation (maximum r values ranged between 0.86 and 0.99).

With respect to the impact of video-based analysis on reconstructing the joint centers trajectories and consequently on the joint moments (comparison h, Table 2 and Table 5 and Figure 8) calculation, overall, an excellent correlation was found between the majority of the trials. However, some trials revealed an inverse correlation mainly in correspondence of both the ankle joint moments and of the hip ab- and adduction moment (only one trial in the case of the knee varus-valgus moment).

Finally with respect to the impact of a reduced marker set in favor of a faster test procedure, results of the last comparison (i) showed an overall excellent correlation (maximum r > 0.94), even though some trials also displayed an inverse correlation in this case in correspondence of both the ankle dorsi-plantarflexion moments and the hip ab- and adduction moment (only one trial in the case of the knee varus-valgus moment).

Since joint center positions are used to compute both joint angles and joint moments, the overall good results revealed in terms of both RMSE% and correlation (comparison a, b, h Table 2 and Table 5) support the adoption of video-based analysis for assessing both lower limb joint moments and angles during a task which is often used in the clinical functional assessment. Caution should be paid in the case of plantar pressure insoles as, even though the majority of the trials revealed excellent to good correlation, some trials displayed poor or inverse correlation. In particular it should be taken into account that the ankle joint was identified as the most affected by errors associated with the measurement of the GRF obtained from the plantar pressure insoles. This was found either when the impact of excluding the contribution of both anterior-posterior and medial-lateral GRFs was verified (comparisons e, f, g) or when the vertical component of the GRF was retrieved from the plantar pressure insoles.

In taking into consideration the choice of the examined task, it should be mentioned that in clinical and sport-specific settings the quality of two movements is frequently assessed within preseason screening, the double-leg and the single-leg squats, that are considered more strenuous tasks than gait, but still not as demanding as jump tests [69]. These tasks, assessed by means of electrogoniometers, have been found attractive by clinicians thanks to the short time required for administering the test and the reliability of the measures of lower extremity function that can be provided with limited equipment. In some cases, measures of lower limb muscle activity were also included by means of surface electromyography [70]. During single and double leg squats, altered movement patterns were detected on subjects suffering from knee-complaints, such as poor alignment, implying excessive medial displacement of the knee in relation to the foot or hip. This alteration is usually associated with ACL injury since it can cause an increased risk for re-injuries during side cut maneuvers. Furthermore, double leg squat exercises are used in early rehabilitation of ACL injured individuals to strengthen quadriceps and hamstring muscles and to inform treatment selection [71].

Certain limitations can be found and should be acknowledged.

Regarding the instrumentation, in this study, Pedar X plantar pressure data were used according to manufacturer’s indications [41] in agreement with current literature [66,72,73]. However a publication by [74] indicated the possibility of improving the performance of the instrument by applying a deconvolution-based algorithm to compensate for the sensor crosstalk effects. Future developments might imply the adoption of this algorithm and the assessment of the impact on the calculation of the joint kinetics.

First of all, only one task was examined among the different ones which are objects of observation within the different clinical screening tasks, such as the Functional Movement Screen, Y Balance/Star Excursion Balance Test, Tuck Jump Assessment, Drop Jump Screening Test and the Landing Error Scoring System. The similarities between a landing and a squat, which both involve descending and ascending phases, should be considered together with the fact that the lower external loading applied during squats is considered safer compared with landings, and this represents a crucial aspect when assessing post-injury subjects [75]. Future developments might include verifying suitability of the proposed technique in more challenging tasks, such as side cut maneuvers, drop landing or drop jump, single-leg squat or squat jumps. Indeed, unilateral landing involving excessive knee abduction has been identified as one of the most frequent actions associated with the incidence of ACL injuries [21]. However, in pursuing this further objective the role played by shear forces in these tasks should be taken into account, as plantar pressure measures are involved.

In terms of reliability analysis, Pearson correlation coefficient was chosen as an alternative to CMC by considering that computing the CMC over time series with limited range and great standard deviation should be avoided [30]. For similar reasons ICC was also not applied. In considering the large variation across trials reported in this study, it should be noted that CMC and ICC measures of correlation among trials are usually applied in the context of gait analysis where smaller differences between subjects are expected for one of the most frequent tasks performed during our daily living activities [30,31]. A larger between subjects variability is expected for the squat task analyzed within this contribution. Finally the most recent state of the art reported the adoption of machine learning to retrieve joint kinetics during sport activities by means of IMU sensors [76]. Interestingly very good agreement with standard gait lab measures during the first landing phase of the Vertical Drop Jump was found. Future work could imply extending ACL Quick Check^®^ technique to other tasks.

## 5. Conclusions

When considering the overall results of the present study, lower impact of the video-based technology was revealed on the joint moment computation with respect to other sources of errors, such as the exclusion of the shear forces from the plantar pressure insoles measures or the fact that the “normal force” is derived from the pressure measured from an array of sensors. It should be mentioned that in most cases lower RMSE% values were recorded when comparing the joint moments retrieved by means of stereophotogrammetry and force plates with the ones obtained through video analysis and plantar pressure insoles. Correlation analysis results suggest that caution should be paid when applying this methodology in the presence of important shear forces exchanged between the foot and the insole. Different options for the estimation of intersegmental moments were presented, and of them, none should be considered correct or incorrect, but each one should be assessed in the specific context of use.

In the context of clinical screening tests for ACL injury prevention, easily applicable tools should be adopted that provide a quantitative assessment of the neuromuscular and biomechanical deficits associated with the risk of injury [23]. The present contribution could represent a compromise between the need of repeatable quantitative measures to predict injury and guide injury prevention programs/training and the requirement to apply clinical movement screening within the constraints of preseason tests (i.e., short time required, low complexity in the execution, applicable on field). Nevertheless, it should be mentioned that reliability of clinically accepted screening tools for injury prevention [17,18,19,20,21,22] might be compromised when scores are assigned by different clinicians. Therefore, the present methodology could be used in combination with the already available screening tools in order to provide more consistent results.

## Figures and Tables

**Table 1:** Demographic data of the HS.

Dataset	Age (Years) Mean (SD)	Weight (kg) Mean (SD)	Height (m) Mean (SD)	BMI (kg/m^2^) Mean (SD)	Shoe Size Mean (SD)
HS	26.6 (3.66)	66.7 (10.84)	1.73 (0.07)	22.04 (2.28)	40.4 (2.27)

**Table 2:** Results of the comparison between joint center trajectories and joint sagittal angles based on the markers reconstructed with stereophotogrammetry (STF) and those reconstructed through Track On Field (TOF): RMSE% and Pearson/Spearman’s correlation coefficient. R = right; L = left; H = hip; K = knee; A = ankle; JC = joint center; ML = medial lateral direction; V = vertical direction; AP = anterior posterior direction.

	STF Mean (SD) (mm) or (Degrees)	TOF Mean (SD) (mm) or (Degrees)	RMSE% of STF (Min < Mean < Max)	r (Pearson/Spearman) (Min < Mean < Max)
RHJC-ML	−8.04 (13.10)	−8.67 (14.75)	(2.63 < 18.29 < 25.41)	(−0.46 < 0.54 < 1)
RHJC-V	−190.27 (55.58)	−184.28 (56.70)	(0.92 < 7.16 < 12.31)	(0.84 < 0.97 < 1)
RHJC-AP	−88.38 (40.88)	−85.12 (36.37)	(1.01 < 11.65 < 16.52)	(0.27 < 0.93 < 1)
LHJC-ML	−8.28 (13.52)	−9.03 (14.74)	(2.58 < 17.19 < 23.64)	(−0.48 < 0.55 < 1)
LHJC-V	−188.31 (55.47)	−181.40 (55.85)	(0.84 < 7.33 < 13.25)	(0.83 < 0.97 < 1)
LHJC-AP	−88.44 (37.26)	−84.10 (35.16)	(1.20 < 13.65 < 18.25)	(0.3 < 0.93 < 1)
RKJC-ML	−25.06 (12.20)	−24.38 (20.75)	(1.34 < 16.46 < 21.59)	(0.67 < 0.9 < 1)
RKJC-V	−44.93 (24.71)	−40.90 (12.92)	(0.74 < 5.92 < 8.74)	(0.84 < 0.97 < 1)
RKJC-AP	105.48 (27.03)	96.80 (33.29)	(1.19 < 10.52 < 18.80)	(0.82 < 0.95 < 1)
LKJC-ML	34.56 (10.68)	31.78 (23.61)	(1.73 < 12.93 < 17.41)	(0.65 < 0.95 < 1)
LKJC-V	−43.30 (24.59)	−39.31 (12.17)	(0.90 < 6.66 < 9.96)	(0.87 < 0.97 < 1)
LKJC-AP	105.00 (28.70)	94.55 (30.49)	(1.26 < 10.45 < 18.16)	(0.82 < 0.96 < 1)
RAJC-ML	2.61 (1.42)	2.70 (2.78)	(1.13 < 12.38 < 16.04)	(0.28 < 0.85 < 0.99)
RAJC-V	−1.81 (0.87)	−1.93 (1.20)	(2.39 < 25.66 < 35.75)	(−0.1 < 0.68 < 0.98)
RAJC-AP	7.58 (2.64)	6.80 (2.83)	(1.35 < 13.67 < 18.52)	(0.54 < 0.9 < 0.99)
LAJC-ML	−0.79 (0.88)	−0.58 (2.63)	(1.78 < 12.52 < 15.73)	(0.26 < 0.68 < 0.99)
LAJC-V	−0.95 (0.52)	−1.23 (1.25)	(6.16 < 48.60 < 61.84)	(0.13 < 0.64 < 0.96)
LAJC-AP	7.78 (2.98)	7.02 (3.29)	(2.05 < 17.33 < 23.55)	(0.71 < 0.91 < 0.99)
Hip—Flexion-Extension	65.99 (20.02)	73.34 (21.19)	(8.89 < 11.20 < 13.79)	(0.77 < 0.96 < 1)
Knee—Flexion-Extension	70.85 (25.67)	69.88 (26.72)	(4.13 < 6.83 < 9.98)	(0.86 < 0.97 < 1)
Ankle—Flexion-Extension	25.91 (8.47)	24.35 (8.97)	(6.48 < 9.54 < 15.59)	(0.87 < 0.97 < 1)

**Table 3:** Results of the comparison between the vertical component of GRF recorded through the force plates and the plantar pressure system: RMSE% and Pearson/Spearman’s correlation coefficient.

	Bertec Mean (SD)	Novel Mean (SD)	RMSE% of Bertec (Min < Mean < Max)	r (Pearson/Spearman) (Min < Mean < Max)	% of Tests with *p* < 0.05
GRF (%BW)—Vertical Component	53.05 (4.33)	35.02 (6.9)	(27.71 < 30.61 < 32.6)	(0.11 < 0.37 < 0.86)	86%

**Table 4:** Results of the comparison between the COP displacement recorded through the force plates and the plantar pressure system: RMSE% and Pearson/Spearman’s correlation coefficient. Statistically significant values are reported as *p* < 0.05. ML = medial/lateral, AP = anterior/posterior.

	Bertec Mean (SD)	Novel Mean (SD)	RMSE% of Bertec	r (Pearson/Spearman)	*p* (*p* < 0.05)
COP Range ML (mm)	14.65 (6.22)	6.26 (4.07)	33.14	0.6	*p* < 0.001
COP Range AP (mm)	55.85 (19.16)	46.5 (23.87)	24.6	0.47	*p* < 0.001
COP SD ML (mm)	5.3 (1.71)	3.14 (1.15)	31.53	0.96	*p* < 0.001
COP SD AP (mm)	26.05 (8.14)	24.29 (6.95)	9.07	0.94	*p* < 0.001
COP path length ML (mm)	30.44 (9.15)	12.94 (7.37)	35.84	0.49	*p* < 0.001
COP path length AP (mm)	143.16 (35.67)	101.85 (53.21)	29.42	0.3	*p* < 0.05

**Table 5:** Results of the comparisons between the different methods for the calculation of joint moments (points from e to i in the method section).

	Joint Moments (% BWx H)	Hip Flex/Extension	Hip Add-Abduction	Knee Flex-Extension	Knee Varus-Valgus	Ankle Dorsi-Plantarflexion	Ankle Inv-Eversion
ACL Quick Check^®^ integrated	Mean (SD)	5.81 (2.32)	0.44 (1.87)	3.08 (1.15)	1.17 (−1.04)	−0.05 (0.62)	−0.28 (0.47)
Inverse Dynamic approach	Mean (SD)	3.15 (1.39)	−0.81 (0.88)	3.88 (0.61)	0.32 (0.77)	0.89 (0.68)	0.04 (0.56)
Ground Reaction Vector 1D	Mean (SD)	4.73 (1.37)	−1.91 (1.3)	3.85 (0.74)	1.12 (1.11)	0.97 (0.73)	0.38 (0.57)
Ground Reaction Vector 3D	Mean (SD)	4.02 (1.69)	−0.25 (1.02)	4.25 (0.68)	0.22 (0.85)	0.91 (0.69)	0.05 (0.58)
ACL Quick Check^®^ integrated STF	Mean (SD)	5.4 (2.17)	−0.77 (1.35)	3.31 (1.11)	0.9 (0.94)	0.22 (0.45)	−0.44 (0.33)
ACL Quick Check^®^	Mean (SD)	5.61 (2.33)	−0.82 (1.53)	3.02 (1.18)	0.91 (0.87)	0.19 (0.44)	−0.39 (0.33)
Comparison e—Inverse Dynamic approach vs. ACL Quick Check^®^ integrated	RMSE% (min < mean < max)	(29.68 < 51.27 < 58.42)	(24.59 < 34.42 < 59.45)	(8.25 < 22.24 < 24.99)	(17.4 < 42.07 < 53.67)	(10.3 < 40.32 < 50.65)	(9.24 < 17.88 < 19.81)
	r (Pearson/Spearman) (min < mean < max)	(0.49 < 0.81 < 0.97)	(−0.83 < 0.44 < 0.96)	(0.65 < 0.9 < 0.99)	(−0.5 < 0.27 < 0.88)	(−0.9 < −0.1 < 0.93)	(−0.76 < 0.34 < 0.93)
	% of tests with *p* < 0.05	98%	93%	100%	83%	87%	83%
Comparison f—Ground Reaction Vector 3D vs. ACL Quick Check^®^ integrated	RMSE% (min < mean < max)	(20.91 < 35.32 < 40.23)	(30.56 < 36.29 < 55.38)	(13.21 < 24.05 < 26.76)	(25.78 < 41.75 < 52.38)	(39.93 < 47.73 < 64.51)	(16.82 < 18.41 < 19.81)
	r (Pearson/Spearman) (min < mean < max)	(0.31 < 0.81 < 0.98)	(−0.8 < 0.33 < 0.94)	(0.68 < 0.91 < 0.99)	(−0.47 < 0.26 < 0.88)	(−0.91 < −0.11 < 0.85)	(−0.79 < 0.34 < 0.92)
	% of tests with *p* < 0.05	100%	87%	100%	73%	83%	87%
Comparison g—Ground Reaction Vector 1D vs. ACL Quick Check^®^ integrated	RMSE% (min < mean < max)	(15.64 < 30.33 < 35.57)	(39.62 < 44.43 < 58.36)	(13.25 < 21.26 < 25.5)	(32.75 < 35.62 < 45.92)	(40.05 < 47.55 < 64.11)	(22.99 < 25.89 < 26.73)
	r (Pearson/Spearman) (min < mean < max)	(0.46 < 0.83 < 0.98)	(−0.9 < 0.39 < 0.96)	(0.63 < 0.92 < 0.99)	(−0.8 < 0.26 < 0.95)	(−0.92 < −0.11 < 0.87)	(−0.68 < 0.23 < 0.86)
	% of tests with *p* < 0.05	100%	83%	100%	75%	83%	83%
Comparison h—ACL Quick Check^®^ integrated vs. ACL Quick Check^®^ integrated STF	RMSE% (min < mean < max)	(4.73 < 6.88 < 8.21)	(6.18 < 10.06 < 14.39)	(7.02 < 11.9 < 14.77)	(7.1 < 8.87 < 10.53)	(11.14 < 26.02 < 36.17)	(9.78 < 23.03 < 27.3)
	r (Pearson/Spearman) (min < mean < max)	(0.35 < 0.88 < 1)	(−0.97 < 0.44 < 0.98)	(0.63 < 0.94 < 1)	(−0.36 < 0.73 < 0.99)	(−1 < 0.4 < 1)	(−0.98 < 0.59 < 1)
	% of tests with *p* < 0.05	100%	92%	100%	97%	89%	89%
Comparison i—ACL Quick Check^®^ vs. ACL Quick Check^®^ integrated	RMSE% (min < mean < max)	(1.54 < 3.99 < 5.23)	(24.31 < 54 < 61.6)	(2.98 < 7.12 < 8.37)	(3.21 < 9.74 < 13.51)	(30.18 < 50.24 < 62.67)	(21.95 < 27.91 < 33.77)
	r (Pearson/Spearman) (min < mean < max)	(0.92 < 0.99 < 1)	(−0.97 < 0.41 < 0.94)	(0.96 < 1 < 1)	(−0.59 < 0.7 < 0.99)	(−1 < 0.25 < 1)	(−0.8 < 0.55 < 1)
	% of tests with *p* < 0.05	100%	94%	100%	92%	100%	91%

**Table 6:** Inter-rater repeatability analysis for the joint centers trajectories (top) and joint sagittal angles (bottom) computed with TOF.

	Op 1 Mean (SD) (mm)	Op 2 Mean (SD) (mm)	Op 3 Mean (SD) (mm)	RMSE% (Min < Mean < Max)	CMC
HJC-ML-r	20.5 (0.65)	20.35 (0.86)	19.89 (0.67)	(0.79 < 2.68 < 5.21)	0.9703
HJC-V-r	78.75 (2.83)	74.89 (2.28)	76.69 (2.17)	(0.69 < 3.8 < 7.85)	0.9683
HJC-AP-r	−4.47 (1.32)	−4.13 (1.09)	−4.83 (1.1)	(1.96 < 6.54 < 12.4)	0.9687
Knee center-ML-r	30.61 (0.44)	30.57 (0.39)	30.43 (0.43)	(0.36 < 0.87 < 5.39)	0.9687
Knee center-V-r	44.43 (0.96)	44.71 (1.06)	44.02 (0.86)	(0.74 < 1.35 < 3.13)	0.9688
Knee center-AP-r	39.13 (1.47)	40.57 (1.2)	39.88 (1.2)	(0.97 < 2.67 < 9.52)	0.9694
Ankle center-ML-r	25.82 (0.13)	25.98 (0.2)	25.48 (0.41)	(0.91 < 1.41 < 3.15)	0.9694
Ankle center-V-r	10.41 (0.08)	10.62 (0.09)	10.47 (0.14)	(0.85 < 1.52 < 3.07)	0.9694
Ankle center-AP-r	19.6 (0.14)	19.69 (0.28)	19.8 (0.35)	(0.99 < 1.65 < 4.18)	0.9694
HJC-ML-l	−52.49 (0.66)	−52.74 (0.9)	−52.18 (0.7)	(0.56 < 1.19 < 3.68)	0.9694
HJC-V-l	80.45 (2.66)	77.02 (2.27)	78.59 (2.08)	(0.47 < 3.46 < 7.12)	0.9706
HJC-AP-l	−8.25 (1.37)	−7.66 (1.53)	−8.66 (1.27)	(2.25 < 6.45 < 11.06)	0.9708
Knee center-ML-l	−51.55 (0.25)	−51.36 (0.36)	−51.35 (0.34)	(0.17 < 0.55 < 2.04)	0.9708
Knee center-V-l	45.27 (0.79)	45.4 (0.94)	44.78 (0.78)	(0.65 < 1.18 < 3.29)	0.9709
Knee center-AP-l	37.01 (1.6)	38.54 (1.17)	37.93 (1.24)	(1.09 < 3.36 < 10.29)	0.9708
Ankle center-ML-l	−53.96 (0.09)	−53.93 (0.22)	−53.5 (0.32)	(0.4 < 0.77 < 2.48)	0.9708
Ankle center-V-l	10.49 (0.17)	10.52 (0.09)	10.44 (0.1)	(0.79 < 1.29 < 2.69)	0.9708
Ankle center-AP-l	19.3 (0.23)	19.08 (0.18)	19.89 (0.59)	(2.25 < 3.18 < 6.28)	0.9708
Hip-FE-r	67.29 (4.06)	61.86 (2.99)	68.85 (2.36)	(3.07 < 7.37 < 11.28)	0.9622
Knee-FE-r	75 (5.09)	80.06 (3.97)	78.05 (3.58)	(2.38 < 4.09 < 7.43)	0.9834
Ankle-FE-r	28.58 (2.72)	30.8 (2.13)	32.29 (4.06)	(4.47 < 7.63 < 14.94)	0.9781
Hip-FE-l	67.28 (4.19)	61.28 (3)	67.49 (2.31)	(3.07 < 6.92 < 11.34)	0.976
Knee-FE-l	69.87 (4.88)	74.96 (3.4)	73.09 (3.13)	(1.36 < 4.93 < 12.77)	0.9786
Ankle-FE-l	25.36 (2.74)	28.03 (1.6)	30.39 (1.95)	(4.56 < 9.59 < 26.74)	0.9784

**Figure A1:**
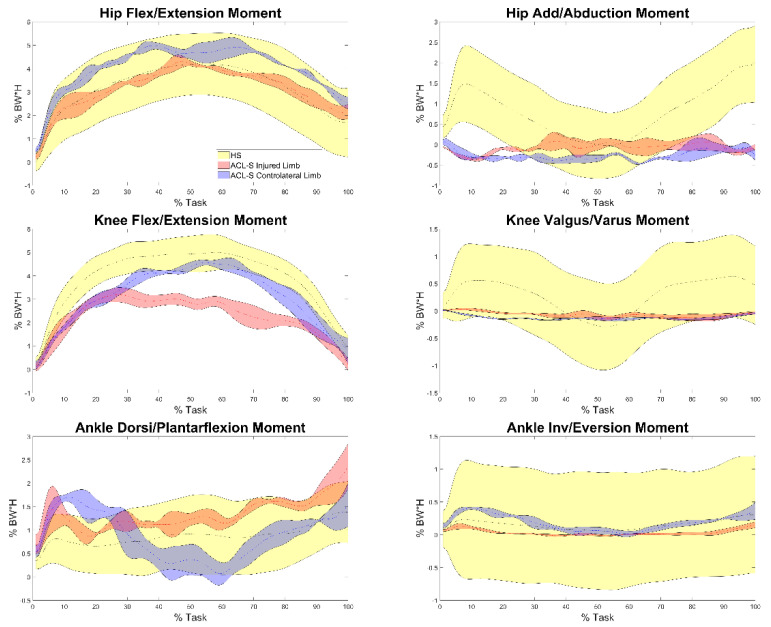
Hip, knee and ankle adduction/abduction and flexion/extension moments, calculated through inverse dynamics: HS (yellow), ACL-S injured limb (red) and ACL-S contralateral limb (blue). Average plus and minus one standard deviation are reported as coloured bands. Average plus and minus one standard deviation are reported as coloured bands.

**Figure A2:**
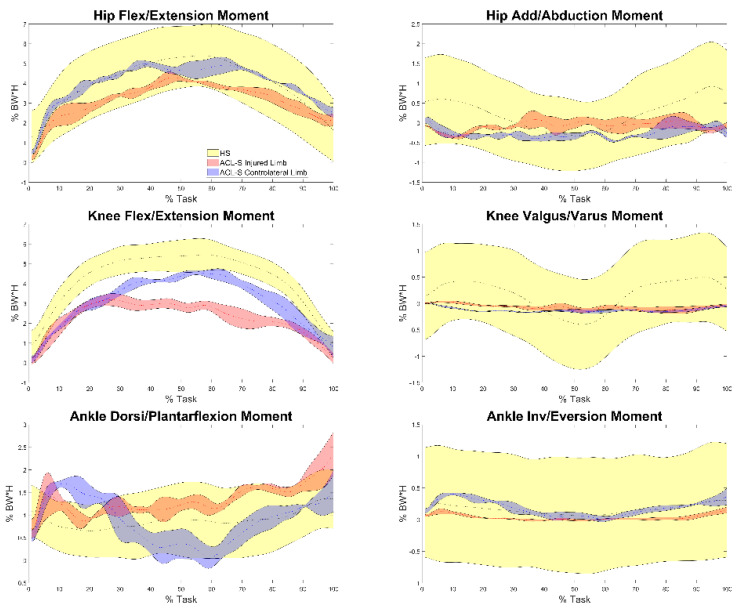
Hip, knee and ankle adduction/abduction and flexion/extension moments, calculated through the Ground Reaction Vector approach: HS (yellow), ACL-S injured limb (red) and ACL-S contralateral limb (blue). Average plus and minus one standard deviation are reported as coloured bands. Average plus and minus one standard deviation are reported as coloured bands.

**Figure A3:**
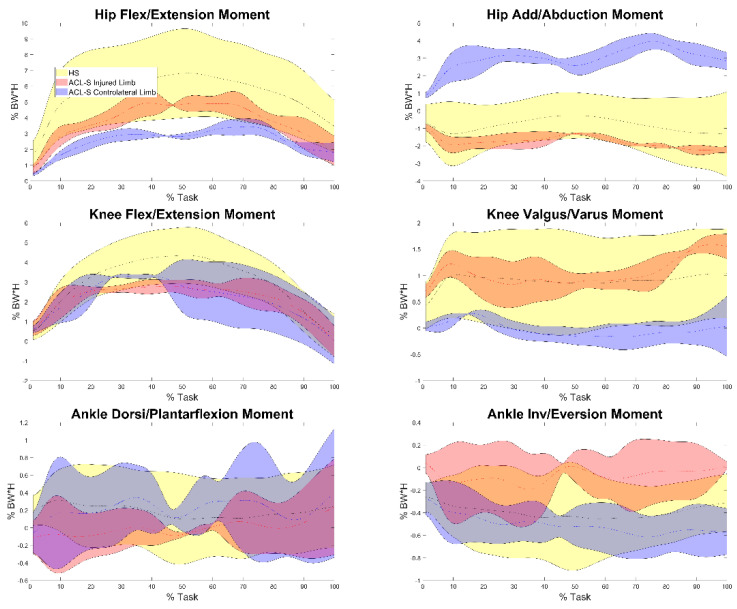
Hip, knee and ankle adduction/abduction and flexion/extension moments, calculated through the ACL Quick Check^®^ integrated: HS (yellow), ACL-S injured limb (red) and ACL-S contralateral limb (blue). Average plus and minus one standard deviation are reported as coloured bands. Average plus and minus one standard deviation are reported as coloured bands.

**Figure A4:**
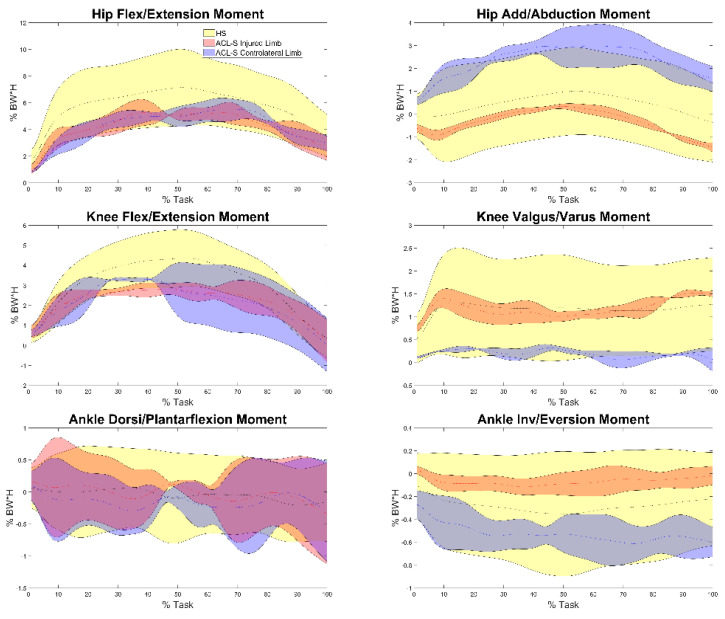
Hip, knee and ankle adduction/abduction and flexion/extension moments, calculated through theACL Quick Check^®^: HS (yellow), ACL-S injured limb (red) and ACL-S contralateral limb (blue). Average plus and minus one standard deviation are reported as coloured bands. Average plus and minus one standard deviation are reported as coloured bands.

**Figure A5:**
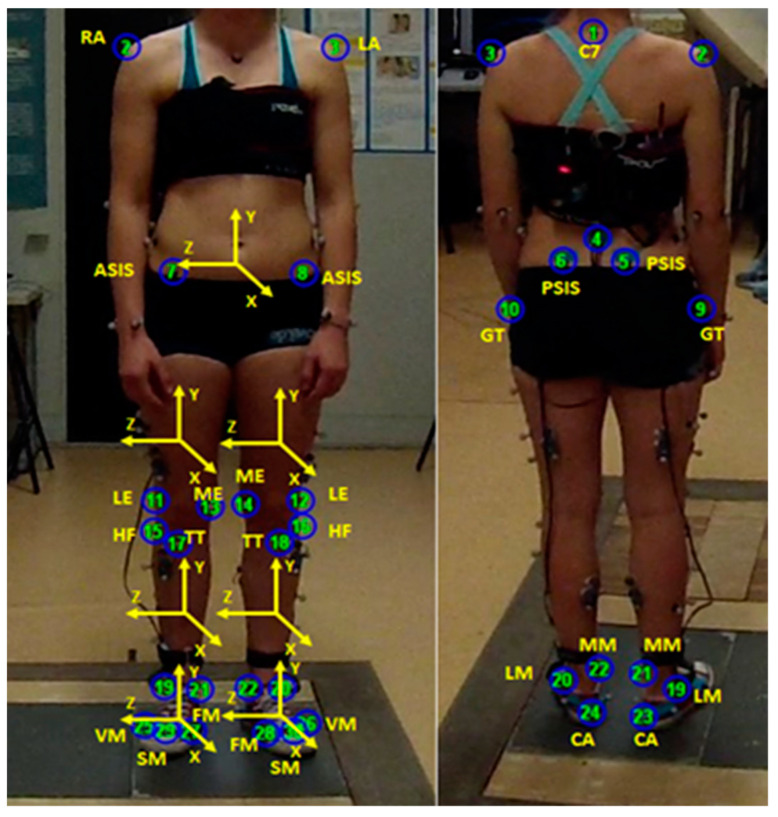
Anatomical landmarks position and anatomical reference frames for the pelvis, thigh, shank.

## Data Availability

Data supporting reported results can be provided upon request to the corresponding author.

## Appendix A. Case Study

One subject who suffered an ACL rupture (from now on referred to as ACL subject (ACL-S)) acquired 4 months after the ACL surgery reconstruction (i.e., semitendinosus and gracile) was included as a case study. Demographic data are reported in Table A1.

**Table A1:** Demographic data of the ACL-S.

Dataset	Age [Years] Mean (SD)	Weight [Kg] Mean (SD)	Height [m] Mean (SD)	BMI [Kg/m^2^] Mean (SD)	Shoe Size Mean (SD)
ACL-S	27	68	1.77	21.7	42

The data analysis of the ACL-S variables was performed as for the HS subjects.

In comparing the results of the ACL-S with the HS only a descriptive statistics was used, hence the data of 3 trials were averaged and represented in terms of mean ± 1 SD for what concerns the ACL-S, while the data of 30 trials (3 trials for each subject) were averaged and represented in terms of mean ± 1 SD for the HS (normative bands, Figure A1, Figure A2, Figure A3 and Figure A4). It should be noted that similar consideration can be driven from the comparison between the ACL-S and the HS for what concerns the hip, knee and ankle flexion-extension moments computed with all the methodologies with the only exception of ACL Quick check^®^ integrated that showed lower values of the hip flexion-extension moment of the contralateral limb for the ACL-S in comparison with the HS (Figure A3). When considering the coronal plane, the hip ab-adduction moment of the ACL-S exceeded the normative bands by 5% BWxH and 2% BWxH respectively when computed by ACL Quick check^®^ integrated or ACL Quick check^®^ (Figure A3 and Figure A4). This difference was not detected by the other methodologies.

By considering that the comparison between healthy and pathologic cohorts of subjects was out of the scope of the present manuscript, only one pathologic subject was included, thus preventing the possibility to define a specific cut point for injury. Further studies could include the definition of a cut point through the comparison between a cohort of post injured subjects at different time frames of their rehabilitation programs.

## Appendix B

The maximum error due to linearity or hysteresis is 0.2% of the full-scale output signal. The calibration matrix is already stored in the force plate thus all outputs are calibrated and corrected for any cross talk. Sensitivity is 5 V per rated output. Resolution of output signal is at least 0.02% of full scale. Operating temperature range is 0–50 °C.

**Table A2:** Force plate technical specifications (Bertec Corp., FP4060-08) [39].

Model	Size (mm)			Weight (kg)	Rated Load (kN)		Natural Frequency (hz)		
	L	W	H		Fz	Fx, Fy	Fz	Fx	Fy
4060-08	600	400	83	28	10	5	340	550	540

**Table A3:** Plantar pressure insoles technical specifications (Novel gmbh, PedarX) [40].

Shoe Size	36 to 46 (European)
thickness (mm)	1.9
number of sensors	99
pressure range (kPa)	15–600
hysteresis (%)	<7
resolution (kPa)	2.5
measurement frequency (Hz)	100
offset temperature drift (kPa/K)	<0.5
minimal bending radius (mm)	20

**Table A4:** Motion capture system technical specifications (BTS srl, SMART-D) [34].

	DX 400
Infrared digital cameras per datastation	up to 16
Sensor Resolution	1 Mpixel
Acquisition frequency at maximum resolution	100 fps
Maximum acquisition frequency	300 fps
Accuracy/Volume	<0.3 mm on 4 × 3 × 3m

Video tracking (Track on Field, BBSoF srl).

For the video acquisitions, 4 GoPro Hero3 and 4 GoPro Hero7 (1080 × 1920 pixel resolution, 30 fps) were used.

Please refer to [47] or the validation of the video tracking software.

## Appendix C

### Appendix C.1. Joint Moment Calculation Following the “Ground Reaction Vector” Approach

The joint moment calculation, referred to as the “ground reaction vector” approach according to [55], is a simplified method which consists of multiplying the ground reaction force vector by its moment arm at each joint [59], without any assumptions on segment accelerations and/or the body segment inertial parameters.

In particular, the moment arm was calculated as the distance between the joint center and the point of application of the ground reaction force, independently from which type of ground reaction force was considered (i.e., measured through force plates or plantar pressure insoles). It is worth mentioning that the “ground reaction vector approach” is then differentiated for the three anatomical planes: sagittal, frontal and transverse.

(A1) $$M_{i}^{loc}=\left(COP^{loc}-JC_{i}^{loc}\right)\times F_{g}^{glob}=R_{glob}^{loc}\cdot M_{i}^{glob}$$

i *i*th joint (ankle, knee, hip)
loc local reference system at the (ankle, knee, hip)
glob global reference system of the laboratory, the y axes oriented vertically perpendicular to the floor
M joint moment at the *i*th joint in the local or global reference system
COP point of application of the ground reaction force vector
JC joint center of the *i*th joint
F ground reaction force vector
Rglobloc rotation matrix that convert a vector expressed in the global reference system to the *i*th local reference system

According to [56] this approach neglects the contribution of the segment inertial and gravitational forces, assuming all the mass concentrated in the body centre of mass, and provides an estimate of joint moments only during the stance phase of gait (when GRF is measured) with minimal computational effort. Despite of its extreme simplicity, this approach has been applied in clinical gait analysis studies, and provided reasonable estimations of joint loadings during the stance phase, when the inertial contribution is minor particularly for distal joints (ankle and knee) [57].

For the slow nature of the studied task (up to a horizontal velocity of 5 m/s [57]), it can be assumed that the momentum with respect to the segment’s centre of gravity can be neglected for all joints of the lower extremity [57,58].

Due to the slow nature of the squat task, in this study this method was adopted for comparison purposes.

### Appendix C.2. Joint Moment Calculation Following the Inverse Dynamic Approach

Inverse dynamics procedure is used to determine the forces that produced a given motion. The inverse dynamic approach adopted in this paper is the Newton-Euler method described in [49,59] according to the following equations:

Equation (A1) calculates the reaction forces at the proximal end of the segment in the global reference system (GRS):

(A2a) $$\sum F_{X}=ma_{X}\;\mathrm{or}\;R_{XP}-R_{XD}=ma_{X}$$

(A2b) $$\sum F_{Y}=ma_{Y}\;\mathrm{or}\;R_{YP}-R_{YD}-mg=ma_{Y}$$

(A2c) $$\sum F_{Z}=ma_{Z}\;\mathrm{or}\;R_{ZP}-R_{ZD}=ma_{Z}$$

where $a_{X}$, $a_{Y}$, $a_{Z}$ are the segment center of mass accelerations in the *X*, *Y*, *Z* GRS directions and $R_{XP}$, $R_{XD}$, $R_{YP}$, $R_{YD}$, $R_{ZP}$, $R_{ZD}$ are the proximal and distal reaction forces in the *X*, *Y*, and *Z* axes.

Equation (A2) transforms both proximal and distal reaction forces into the anatomical axes *x*, *y*, *z*: $R_{XP}$, $R_{XD}$, $R_{YP}$, $R_{YD}$, $R_{ZP}$, $R_{ZD}$ using the global to anatomical matrix transformation based on *θ*_1_, *θ*_2_, and *θ*_3_ rotation angles (*s_i_* = *sin*(*θ_i_*), *c_i_* = *cos*(*θ_i_*)).

(A3) $$\left[\begin{array}{c} x_{3}\\ y_{3}\\ z_{3} \end{array}\right]=\left[\begin{array}{ccc} c_{2}c_{3} & s_{3}c_{1}+s_{1}s_{2}c_{3} & s_{1}s_{3}+c_{1}s_{2}c_{3}\\ -c_{2}s_{3} & c_{1}c_{3}+s_{1}s_{2}s_{3} & s_{1}c_{3}+c_{1}s_{2}s_{3}\\ s_{2} & -s_{1}c_{2} & c_{1}c_{2} \end{array}\right]\left[\begin{array}{c} x_{0}\\ y_{0}\\ z_{0} \end{array}\right]$$

Equation (A3) transforms the distal moments from those previously calculated in the GRS into the ones calculated with respect to the anatomical axes, based on *θ*_1_, *θ*_2_, and *θ*_3_ rotation angles, from now on referred to as: $M_{xd}$, $M_{yd}$, $M_{zd}$.

Equations (A4) reports, for each of the joints, the rotational equations of motion as follows

(A4a) $$I_{x}\alpha_{x}+\left(I_{z}-I_{y}\right)\omega_{y}\omega_{z}=\sum M_{x}=R_{zd}I_{d}+R_{zp}I_{p}+M_{xp}-M_{xd}$$

(A4b) $$I_{\mathrm{xy}}\alpha_{y}+\left(I_{x}-I_{z}\right)\omega_{x}\omega_{z}=\sum M_{y}=M_{yp}-M_{yd}$$

(A4c) $$I_{z}\alpha_{X}+\left(I_{y}-I_{x}\right)\omega_{x}\omega_{y}=\sum M_{z}=-R_{xd}I_{d}-R_{xp}I_{p}+M_{zp}-M_{zd}$$

Ix, Iy, Iz moments of inertia about *x–y–z* axes
ωx, ωy, ωz components of angular velocity about *x–y–z* axes
αx, αy, αz components of angular acceleration about *x–y–z* axes
Mxd, Myd, Mzd previously transformed distal moments about *x–y–z* axes
Rxd, Rxp, Ryd, Ryp, Rzd, Rzp previously transformed joint reaction forces about *x–y–z* axes
Ip, Id distances from center of mass to proximal and distal joints

The unknown variables in these three equations are the three moments ($M_{xp}$, $M_{yp}$ and $M_{zp}$) about the proximal *x, y, z* axes.

### Appendix C.3. Joint Moment Calculation Following the ACL Quick Check® Approach

ACL Quick check^®^ methodology allows the calculation of 3D joint moments, based on plantar pressure data acquired with pressure insoles and 3D joint centres trajectories derived from anatomical landmarks trajectories reconstructed through a tracking of video software. This methodology is patented and University of Padova presently holds the patent (N. 102017000046512). In the present paper the software Track on Field (BBSoF S.r.l.) was used as tracking of feature software, and the Pedar X system (Novel GmbH) as plantar pressure system. With respect to ACL Quick Check methodology a commercial version is available as a plug-in of the software Track on Field (BBSoF S.r.l.), raw plantar pressure data and 3D anatomical landmarks trajectories are required as input.

### Appendix C.4. Video Based Reconstruction of Anatomical Landmark Points

The 3D coordinates of the points identified on the body of the subject were reconstructed with the software Track on Field (BBSoF S.r.l.) [47].

### Appendix C.5. Joint Angles Calculation

Iorgait markerset [42] (Figure 1 in the paper and Figure A5) was adopted for joint angles calculations. The paper [42] proposes the definition of the anatomical reference frames (Figure A5) and joint rotations according to international recommendations [51,52].

Standard coordinate systems are adopted for each joint, which entail defining flexion/extension as the relative rotation about an axis taken as the medio-lateral axis (Z) of the proximal segment; internal/external rotation as the relative rotation about the vertical axis (Y) of the distal segment; and abduction/adduction as the relative rotation about a ‘floating’ axis (X) orthogonal to these two at each collected sample [53]. This terminology is adopted for the hip and knee joints, while for the ankle joint the three rotations are referred to as dorsiflexion-plantarflexion, inversion/eversion, and abduction/adduction [42].

Flexion/extension (or dorsi/plantarflexion) joint angles only were considered for the purpose of the present study.
